# Supplementary material for: ZNF16 is a nucleolar-associated protein that regulates expression of rDNA and cancer-associated genes
Source: Biol Open. 2026 Jan 14;15(1):bio062336. doi: 10.1242/bio.062336 (PMC12833810; doi:10.1242/bio.062336)
Supplement: Supplementary information [file biolopen-15-062336-s1.pdf]

Table S1. RNA-seq read summary and differential expression results.

Available for download at
https://journals.biologists.com/bio/article-lookup/doi/10.1242/bio.062336#supplementary-data

Biology Open • Supplementary information
